# Association between intimate partner violence during pregnancy and risk of preterm birth

**DOI:** 10.1186/s12889-021-11625-8

**Published:** 2021-09-03

**Authors:** Sanni Yaya, Emmanuel Kolawole Odusina, Nicholas Kofi Adjei, Olalekan A. Uthman

**Affiliations:** 1 School of International Development and Global Studies, University of Ottawa, 120 University Private, Ottawa, ON K1N 6N5 Canada; 2 The George Institute for Global Health, Imperial College London, London, UK; 3 Department of Demography and Social Statistics, Federal University, Oye, Ekiti Nigeria; 4 Department of Public Health, Policy and Systems, University of Liverpool, Liverpool, UK; 5 Warwick Centre for Applied Health Research and Delivery (WCAHRD), Division of Health Sciences, Warwick Medical School, University of Warwick, Coventry, CV4 7AL UK

**Keywords:** Women, Intimate partner violence, Preterm birth, Global health, Zimbabwe

## Abstract

**Background:**

Preterm birth is a risk factor for child survival in both the short and long term. In Zimbabwe, the prevalence of preterm birth is rising, and there are growing concerns about the adverse consequences. This study explored the association between intimate partner violence (IPV) during pregnancy and preterm birth in Zimbabwe.

**Methods:**

Using data from the 2015 Zimbabwe Demographic and Health Survey, we applied propensity score matching to estimate the effect of IPV during pregnancy on preterm birth among women of reproductive age (15–49 years). A total of 4833 pregnant women who gave birth during the five years preceding the survey were analysed.

**Results:**

We successfully matched 79 women who were exposed to IPV during pregnancy to 372 unexposed during pregnancy. Using the matched sample, the probability of preterm delivery was significantly higher among women who were exposed to IPV during pregnancy than those who were not exposed. The findings showed that 7 out of 79 (8.9%) of women exposed to IPV during pregnancy experienced preterm delivery, and 11 out of 372 (3.0%) of those who were not exposed to IPV during pregnancy experienced preterm delivery. In the urban areas, those exposed to IPV during pregnancy were almost five times more likely to experience preterm delivery (OR = 4.8, 95% CI 2.0–11.6), but the association was not significantly different among women in rural areas.

**Conclusion:**

The findings showed that women exposed to IPV during pregnancy were at increased risk of preterm birth. Some of the risk factors associated with IPV were urban residence, low economic status and unemployment. Effective policies and programmes are required to address the issue of IPV in Zimbabwe.

## Background

Infant and child mortality are growing health issues globally, particularly in sub-Saharan Africa [1–3]. The complications from preterm birth, including sepsis, periventricular leukomalacia, seizures, infections, feeding difficulties, cerebral palsy, visual and hearing problems and necrotising enterocolitis [1] have led to approximately 16% of all deaths and 35% of deaths of newborn babies globally [1]. Although there have been policies and intervention programmes to tackle and reduce the issue of both preterm birth [4, 5] and infant mortality in many countries [5–7], the desired outcomes have not been achieved [1, 6]. To implement effective interventions, there is the need to understand the factors associated with preterm birth. Reducing and mitigating its adverse consequences is also crucial for achieving Sustainable Development Goal 3, which aims to ensure healthy lives and promote well-being for all ages [7].

The issue of intimate partner violence (IPV) is also a worldwide public health and human rights concern [8–10], and it is a risk factor for several mental and physical health outcomes, including STI/HIV infections, unintended pregnancies, injuries and abortion [11–13]. IPV is pervasive globally [14–16], and it comes in the form of sexual, psychological or emotional aggression [9, 13], usually perpetrated by male partners against female partners [9]. Globally, approximately 35% of all women have experienced domestic violence either by their partners or third parties. According to the World Health Organisation (WHO), about three out of every ten women in a relationship had ever experienced domestic violence [14].

Evidence suggests that IPV is one of the most prevalent forms of gender-based violence during or before pregnancy [10], which may have both short and long term adverse consequences for babies and mothers [15]. Experiences of IPV during pregnancy may increase women vulnerability to ill health [12]. The various forms of IPV, such as physical, psychological, sexual, or emotional abuse, have also been shown to have an indirect adverse effect on foetal health outcomes, including low birth weight [16–18]. For example, a recent study in Ethiopia found an increased risk of preterm birth and low birth weight among pregnant women who experienced multiple forms of IPV [19]. Several risk factors, including younger age, alcohol abuse, past exposure to violence (childhood sexual abuse), marital status and poverty, have been shown to be associated with IPV during pregnancy [20, 21].

A recent study carried out in sixty three low-and middle-income countries also found a negative association between increased acceptability of IPV against women and antenatal care service utilization among women, but there were variations across regions and countries [22–27]. Delay in seeking timely antenatal care has also been associated with IPV during pregnancy [28].

An association between antenatal care service utilization and preterm birth has been found in prior studies [29–32]. Other previous studies suggest that antenatal care service utilization may be useful only in identifying high-risk women but can also facilitate timely intervention [29, 33]. The risk of preterm birth has also been shown to be higher among pregnant women who start antenatal care late than those who start early [34, 35]. Meanwhile, other factors identified to be associated with preterm birth are vaginal bleeding, hypertensive disorder, smoking and stress [36, 37].

Maternal and child mortality is high in sub-Saharan Africa [38, 39], with Zimbabwe having one of the highest rates of maternal health challenges in the region [17]. While the average sub-Saharan rate of maternal mortality was 500/100000 in 2010, Zimbabwe recorded an average of 570/100000 live births [17]. In a recent study in Zimbabwe, Shamu et al. (2018) found intimate partner violence and forced first sex to be associated with adverse health consequences for unborn babies and mothers.

Despite the substantial evidence that IPV during pregnancy impacts negatively on both mother and child health [17], no study has explored the relationship between IPV and preterm birth in Zimbabwe. Hence, this study investigated the relationship between IPV during pregnancy and preterm birth in Zimbabwe. The following research questions will be addressed. (i) Is intimate partner in pregnancy associated with preterm birth in Zimbabwe? (ii) What are the risk factors associated with preterm birth and IPV exposure in Zimbabwe?

## Methods

### Data sources

The data for the analysis was from the latest (2015) Zimbabwe Demographic and Health Survey (ZDHS). The survey was undertaken by the Zimbabwe National Statistics Agency in collaboration with other international organisations, and it is nationally representative of men and women in their reproductive age (15–49 years). In the survey, samples of households were drawn using a stratified multistage cluster sampling design. The standard woman’s questionnaire was used to obtain information on women’s background, reproductive and birth history, information about family planning, maternity history, child immunization, child health and nutrition, marriage and sexual activity, fertility preferences, husband’s background and HIV/AIDS related knowledge and behaviours and other health issues. For this study, we limited our sample to women who gave birth in the five years preceding the survey (*N* = 4833).

### Outcome measure

The outcome variable for this study was preterm birth. Preterm birth was defined as babies born alive before 37 weeks of completed pregnancy or the last babies the respondents had prior to 37 completed weeks of gestation (approximately less than 8 months). On the other hand, term birth was defined as babies born alive after 37 weeks of completed pregnancy or last babies born alive after 37 completed weeks of gestation (9 months or older).

### Independent variable

Intimate partner violence (IPV) was the independent variable of interest. This variable was a combination of at least one type of intimate partner abuse experienced by a woman (i.e., physical, sexual or emotional). In the survey, women were asked a series of questions about whether they were exposed to any of the aforementioned forms of violence. The questions posed were for example, “did your husband/partner ever: slap, push, shake, punch, beat, kick or try to strangle you, throw something on you, threaten you using a harmful object?” These questions were used to derive physical violence. Sexual violence was assessed by the questions “did your husband/partner ever physically force you to have sexual intercourse even when you did not want? Or force you with threats to perform any sexual acts you did not want?” Psychological violence was assessed using questions such as “did your husband/partner humiliate you in front of others, threaten to hurt you or those close to you with harm?” Response categories were: ‘yes’ (coded as 1) or ‘no’ (coded as 0).

### Covariates

Covariates included in the analysis were maternal age, educational level, residence, wealth index, work status, age at first intercourse, partner’s level of education, prenatal care, number of prenatal care visits and cigarette smoking.

### Statistical analysis

Descriptive statistics, propensity score matching and subgroup risk analysis were performed in this study. We used descriptive statistics to show the distribution of respondents by the key variables. Values were expressed as absolute numbers (percentages) and mean (standard deviation) for categorical and continuous variables respectively. All cases in the DHS data were given weights to adjust for differences in probability of selection and to adjust for non-response in order to produce the proper representation. Individual weights were used for descriptive statistics.

For propensity score analysis, we first examined the baseline characteristics of the respondents and estimated standardised differences for all variables before and after matching. A standardised difference of 10% or more is suggestive of imbalance. We used propensity score methods to account for all measured differences in baseline characteristics between respondents that reported exposure to IPV during pregnancy and those without. The exposed group consisted of women who were exposed to IPV during pregnancy and the unexposed group consisted of those who did not experienced violence during pregnancy. The propensity score for this analysis was estimated using logistic regression. We then matched each respondent with IPV with the closest propensity score on a ratio of 1:5 using a nearest neighbour algorithm with no replacement. Furthermore, we calculated the difference in probability of preterm delivery with and without exposure to IPV during pregnancy in the propensity score–matched cohort.

Finally, we examined the risk by different subgroups, i.e. we calculated odds ratio for the association between exposure to IPV during pregnancy and preterm delivery by socioeconomic status. An odds ratio greater than 1 suggests that preterm delivery is more prevalent among those exposed to IPV. Conversely, a value less than 1 indicates that suggests that preterm delivery is less prevalent among those exposed to IPV.

The null hypothesis was tested against a two-sided alternative hypothesis at a significance level of 5%. The data was analysed using Stata version 16 (Stata Corp, College Station, TX, USA).

## Results

### Sample characteristics

Table 1 presents the socio-demographic characteristics of respondents. Overall, a total of 4833 women gave birth in the five years preceding the survey. Approximately 2.7% and 97.3% of women reported preterm births and term births, respectively. The results further showed that about 3.1% of women reported having ever experienced IPV during pregnancy, while most women (96.9%) did not report any experience of IPV during pregnancy. More than one-fourth of the respondents were within the younger age groups. For instance, 25.5% of the women were in the 25–29 years age group. Most women (70.6%) reported having secondary or higher education. The distribution of husbands’ educational attainment followed a similar pattern, where about 78.6% reported having higher education. Regarding economic status, about 52.2% of the respondents indicated they were working, and 36.2% reported to be in the poor household category. More than two-thirds (60.9%) lived in rural areas. A significant number of women (41.0%) had their first intercourse within the age group of 15–17 years. While nearly all (94.5%) of the respondents registered for prenatal care services, about 40.2% attended 0–4 times of antenatal care.

**Table 1:** Socio-demographic characteristics of respondents

Variables	Total *N* = 4833 (n)	%
**Age (Years)**		
15–19	360	7.5
20–24	1165	24.1
25–29	1232	25.5
30–34	1088	22.5
35–39	630	13.0
40–44	302	6.3
45–49	56	1.2
**Level of Education**		
No formal education	46	1.0
Primary	1376	28.5
Secondary plus	3411	70.6
**Residence**		
Urban	1892	39.2
Rural	2941	60.9
**Wealth Index**		
Poor	1747	36.2
Middle	761	15.8
Rich	2325	48.1
**Work status**		
Working	2521	52.2
Not working	2312	47.8
**Husbands’ level of education**		
No formal education	48	1.2
Primary	803	20.2
Secondary plus	3131	78.6
**Age at first intercourse**		
< 15	273	5.7
15–17	1965	41.0
18+	2560	53.4
**Prenatal Care**		
No	263	5.5
Yes	4560	94.5
**Number of antenatal visit**		
0–3 times	1137	23.6
4–7 times	3091	64.1
8–20 times	595	12.3
**Wanted pregnancy**		
No	1734	35.9
Yes	3099	64.1
**Smoke**		
No	4823	99.8
Yes	10	0.2
Preterm birth		
Yes	132	2.7
No	4701	97.3

### Effect of IPV exposure during pregnancy on preterm delivery

The characteristics of the respondents before and after matching are shown in Table 2. There were important baseline differences between respondents with exposure to IPV during pregnancy and those not exposed. Women who were exposed to IPV during pregnancy tended to be middle-aged adults compared those who were not exposed. Those exposed to IPV were more likely to be from richer wealth index category, rural areas and less likely to have attended antenatal care. We successfully matched 79 respondents exposed to IPV during pregnancy to 372 not exposed to IPV during pregnancy. After matching, absolute standardised differences were less than 10% for most of the variables used for the propensity score matching, suggesting an adequate match (Table 2). Using the matched sample, the probability of preterm delivery was significantly higher among women who were exposed to IPV during pregnancy than those who were not exposed (8.86% vs 2.96%, *p* = 0.015). 7 out of 79 (8.86%) of women who were exposed to IPV during pregnancy reported preterm delivery, while 11 out of 372 (2.96%) of those who were not exposed to IPV during pregnancy experienced preterm delivery.

**Table 2:** Standardised Differences across Covariates: before Matching and after Matching

	Before matching				After matching			
	IPV	No IPV	***p***-value	%bias	IPV	No IPV	p-value	%bias
**Age (Years)**								
20–24	14.6	22.8	0.05	−21.1	14.6	17.3	0.596	−7
25–29	24.3	26.4	0.635	−4.8	24.3	25.0	0.898	−1.8
30–34	24.3	24.1	0.968	0.4	24.3	21.2	0.597	7.2
35–39	20.4	13.4	0.041	18.7	20.4	20.8	0.945	−1
40–44	6.8	6.4	0.884	1.4	6.8	6.2	0.866	2.3
45–49	2.9	1.2	0.135	11.8	2.9	2.5	0.865	2.7
**Level of Education**								
Primary	26.2	28.7	0.578	−5.6	26.2	20.6	0.342	12.6
Secondary plus	73.8	71.3	0.578	5.6	73.8	79.4	0.342	−12.6
**Husbands’ level of education**								
Primary	17.5	19.8	0.565	−5.9	17.5	16.3	0.824	3
Secondary plus	81.6	79.1	0.546	6.1	81.6	83.1	0.771	−3.9
**Wealth Index**								
Middle	11.7	15.4	0.297	−11	11.7	14.0	0.619	−6.8
Rich	63.1	47.5	0.002	31.7	63.1	62.7	0.954	0.8
**Work status**								
Not working	51.5	49.3	0.67	4.2	51.5	53.0	0.824	−3.1
**Residence**								
Urban	43.7	61.6	0	−36.4	43.7	41.7	0.779	3.9
**Smoke**								
Cigarette smoking	1.0	0.1	0.032	11.3	1.0	1.2	0.893	−2.6
**Age at first intercourse**								
15–17	33.0	39.9	0.156	−14.4	33.0	30.7	0.721	4.8
18+	63.1	54.8	0.093	17	63.1	66.4	0.622	−6.7
**Prenatal Care**	93.2	94.5	0.577	−5.3	93.2	93.8	0.866	−2.4
**Number of antenatal visits**								
4–7 times	34.0	48.4	0.004	−29.5	34.0	36.5	0.706	−5.2
8–20 times	16.5	12.5	0.228	11.3	16.5	14.8	0.731	5
**Wanted pregnancy**	62.1	68.9	0.143	−14.2	62.1	61.9	0.977	0.4

*The standardised percentage bias. The standardised % bias is the % difference of the sample means in the treatment and control (full or matched) sub-samples as a percentage of the square root of the average of the sample variances in the treated and non-treated groups [30]

### Risk by different subgroups

**Fig. 1:**
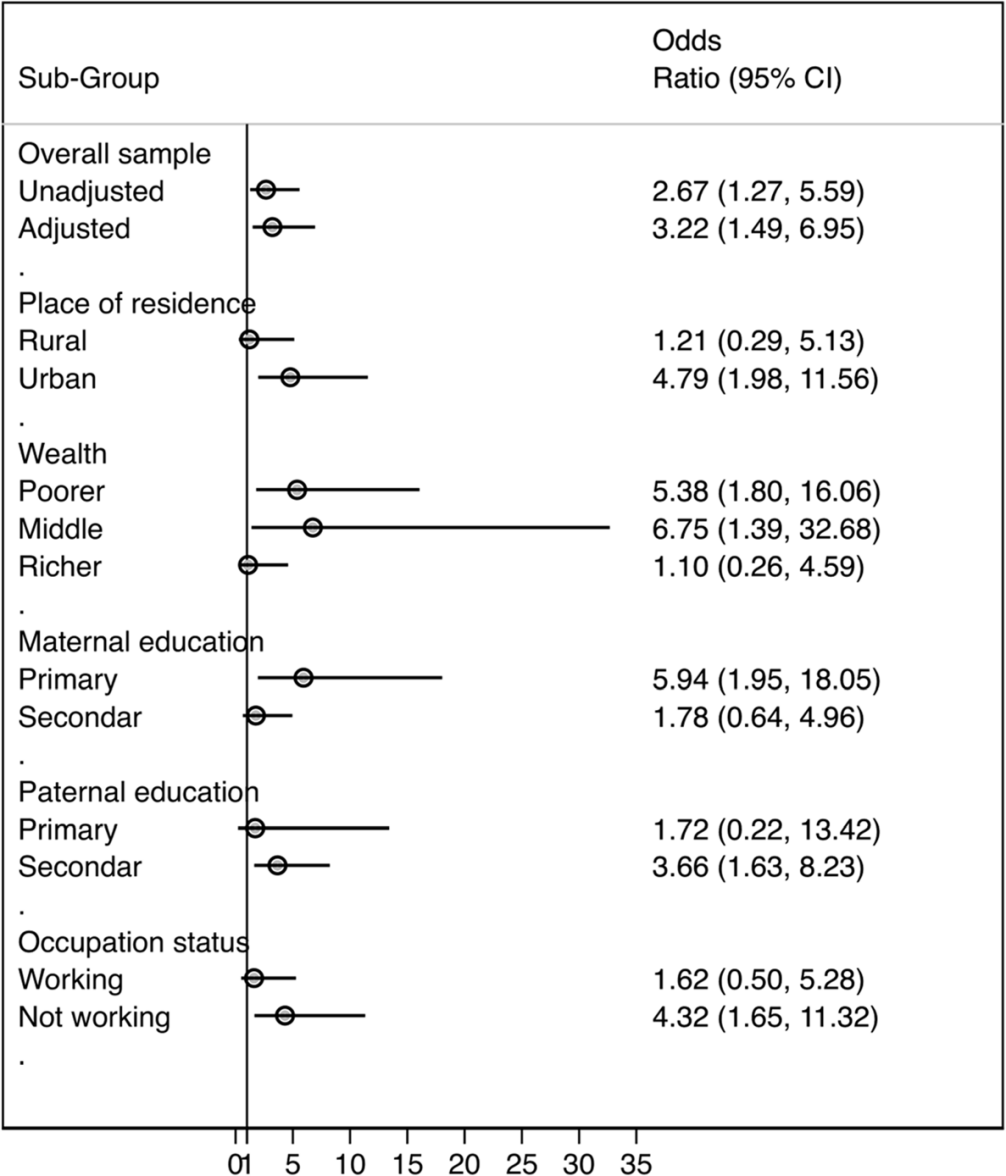
Association between exposure to IPV during pregnancy and preterm delivery

Figure 1 shows the association between exposure to IPV during pregnancy and preterm delivery by socioeconomic status. In the urban areas, the results revealed that women who were exposed to IPV during pregnancy were almost five times more likely to experience preterm delivery (OR = 4.79, 95% CI 1.98–11.56), but the association was not significantly different among women in rural areas. Similarly, the association was significant among women from poorer household, lower education and those who were not working.

## Discussion

Preterm birth is one of the leading causes of complications and death globally among children under 5 years [1, 40]. Approximately 15 million babies are born before completed weeks of gestation (preterm), and the number is on the rise [41, 42]. Prior evidence from WHO estimates from 184 countries with reliable data across the world put the prevalence of preterm birth at 5–18% [42, 43], but there are also country and regional variations [44, 45]. African and Asian countries have the highest rates [46], where more than 60% of preterm births occur in sub-Saharan Africa and South Asia. In sub-Saharan Africa, Zimbabwe has one of the highest prevalence (16.6%) [47].

The prevalence of both IPV [48, 49] and preterm birth [50] are high in Zimbabwe. This study therefore explored the association between intimate partner violence during pregnancy and the risk of preterm birth in Zimbabwe, using DHS data collected in 2015. The analyses involved 4833 women who gave birth during the five years preceding the survey. IPV during pregnancy was the exposure and preterm delivery was the outcome of interest. Overall, 3.1% of women reported having ever experienced IPV during pregnancy; with similar prevalence for preterm births.

We applied propensity score matching to estimate the effect of IPV during pregnancy on preterm birth among women, and successfully matched 79 respondents exposed to IPV during pregnancy to 372 not exposed to IPV during pregnancy. Regarding the characteristics of respondents before and after matching, we found disparities among women who were exposed to IPV during pregnancy and those not exposed. For instance, the findings showed a pattern where women in the middle-aged groups were more likely to be exposed to IPV during pregnancy than older women. This finding is consistent with a recent meta-analyses [51] and previous cross-sectional studies [52, 53]. A possible explanation for this phenomenon is that younger and middle-aged women are more likely to depend on their partners than older women [54, 55], which limits their autonomy in unions [56], leading to excessive control and abuse from their partners.

Consistent with prior studies [9, 57], we found that women who had experienced IPV during pregnancy were more likely to live in rural areas [58, 59], and they had inadequate antenatal visits [9, 57]. These findings may be linked with social norms and traditional belief, particularly in rural areas, where women justify male dominance and abusive acts [60]. Another associated risk factor could be poor socioeconomic conditions including low education and poverty among women in rural areas [58, 59]. Poverty has been shown to be associated with IPV [61, 62], as women who face financial constraints may heavily depend on their partners [63]; and may be at risk of being abused [64]. By contrast, our study showed that rich women were more likely to be exposed to IPV [65]. While this finding is consistent with some previous studies [66], other studies found that improved financial status may be a protective factor against IPV [62, 64, 67]. Nonetheless, we speculate that economically empowered women may also risk being abuse by their male partners who feel threatened by their wealth [68].

The findings revealed more preterm delivery among women who were exposed to IPV during pregnancy compared to those who were not exposed, consistent with prior findings [9, 10, 19, 69, 70]. Nonetheless, a study conducted by Audi et al. (2008) did not find any significant association between domestic violence during pregnancy and prematurity [71]. Our findings further revealed a higher incidence of preterm birth among women who were exposed to IPV during pregnancy in urban areas compared to their counterparts in the rural areas. Women in urban areas were about five times more likely to experience preterm birth during pregnancy. Stress and anxiety of urban life, depression, career and tendency of urban women to marry late are some of the factors that have been implicated to explain this phenomenon [10, 72, 73].

## Limitations and strength

Our study has some limitations. First, the cross-sectional design of this study prevents conclusions that do not permit fortitude of causality between preterm birth and other variables. Second, self-report information by respondents could be affected by recall bias, for instance, errors may occur in reporting of age of respondents at marriage due to undocumented registration of age system Third, the use of propensity score matching does not eliminate or overcome the initial bias in selection [74]. Despite these limitations, this study used nationally representative DHS data, where selected participants were sampled using probability sampling methods. Also, propensity score matching contributes more to precise estimation of treatment response and balances observed baseline covariates between treatment groups [75].

## Conclusion

The findings indicate that exposure to intimate partner violence during pregnancy increased the risk of preterm birth in Zimbabwe. Some of the risk factors associated with IPV were urban residence, low economic status and unemployment. Thus, effective policies and programmes that target women empowerment, education and development are needed to tackle the issue of IPV against women and preterm birth.

## Data Availability

Data for this study were sourced from Demographic and Health surveys (DHS) and available here: http://dhsprogram.com/data/available-datasets.cfm.

## Abbreviations

DHS: Demographic and Health Surveys
IPV: Intimate partner violence
WHO: World Health Organisation
